# Polymer-Coated Nanoparticles for Therapeutic and Diagnostic Non-^10^B Enriched Polymer-Coated Boron Carbon Oxynitride (BCNO) Nanoparticles as Potent BNCT Drug

**DOI:** 10.3390/nano11112936

**Published:** 2021-11-02

**Authors:** Chen-Wei Chiang, Yun-Chen Chien, Wen-Jui Yu, Chia-Yu Ho, Chih-Yi Wang, Tzu-Wei Wang, Chi-Shiun Chiang, Pei-Yuin Keng

**Affiliations:** 1 Department of Material Science and Engineering, National Tsing Hua University, Hsinchu City 300, Taiwan; q9102772@gmail.com (C.-W.C.); chien95837@gmail.com (Y.-C.C.); j0617johnny@gmail.com (C.-Y.H.); h311070@nehs.hc.edu.tw (C.-Y.W.); twwang@mx.nthu.edu.tw (T.-W.W.); 2 Department of Biomedical Engineering and Environmental Science, National Tsing Hua University, Hsinchu City 300, Taiwan; rayboss8@yahoo.com.tw (W.-J.Y.); cschiang@mx.nthu.edu.tw (C.-S.C.)

**Keywords:** BNCT, BCNO, cancer therapy, nanoparticle drug delivery

## Abstract

Boron neutron capture therapy (BNCT) is a powerful and selective anti-cancer therapy utilizing ^10^B-enriched boron drugs. However, clinical advancement of BCNT is hampered by the insufficient loading of B-10 drugs throughout the solid tumor. Furthermore, the preparation of boron drugs for BNCT relies on the use of the costly B-10 enriched precursor. To overcome these challenges, polymer-coated boron carbon oxynitride (BCNO) nanoparticles, with ~30% of boron, were developed with enhanced biocompatibility, cell uptake, and tumoricidal effect via BNCT. Using the ALTS1C1 cancer cell line, the IC_50_ of the PEG@BCNO, bare, PEI@BCNO were determined to be 0.3 mg/mL, 0.1 mg/mL, and 0.05 mg/mL, respectively. As a proof-of-concept, the engineered non-^10^B enriched polymer-coated BCNO exhibited excellent anti-tumor effect via BNCT due to their high boron content per nanoparticle and due to the enhanced cellular internalization and retention compared to small molecular ^10^B-BPA drug. The astrocytoma ALTS1C1 cells treated with bare, polyethyleneimine-, and polyethylene glycol-coated BCNO exhibited an acute cell death of 24, 37, and 43%, respectively, upon 30 min of neutron irradiation compared to the negligible cell death in PBS-treated and non-irradiated cells. The radical approach proposed in this study addresses the expensive and complex issues of B-10 isotope enrichment process; thus, enabling the preparation of boron drugs at a significantly lower cost, which will facilitate the development of boron drugs for BNCT.

## 1. Introduction

More than 50% of cancer patients received radiation therapy during their course of treatment [1]. The combination of chemotherapy and radiation therapy remains the first line of treatment in the clinical management of several solid tumors. However, this treatment paradigm can lead to significant toxicity and side effects in patients because both treatments (i.e., chemotherapy and radiation therapy) destroy the neighboring healthy tissues surrounding the cancer cells or healthy tissues that are in the lane of the radiation beam. Boron neutron capture therapy (BNCT) is an alternative radiotherapy that enables the selective destruction of individual cancer cells when a boron drug is administered. The fundamental principle of BNCT is based on the nuclear reaction between B-10 isotope and a low-energy epithermal neutron to induce lethal damage at the targeted tumor cells. This nuclear reaction produces high-linear energy transfer (LET) particles that dissipates their energy within a very short distance (5–9 µm), which is comparable to the size of a single cell [2,3]. Consequently, BNCT enables the selective destruction of tumor cells, while sparring the adjacent healthy tissues. To date, only two boron drugs (sodium mercaptoundecahydro-closo-dodecaborate (BSH) and borophenylalanine (BPA)) have been approved by FDA for BNCT treatment in patients owing to their low toxicity. BPA/BSH-BNCT have been used for the clinical treatment of various cancers such as hepatic cancer [4], melanoma cancer [5], gastric cancer [6], gliomas [3,7], head, and neck cancer [8,9,10,11,12,13]. However, preclinical and clinical studies on these drugs are still non-conclusive. One of the major challenges of BCNT is the development of a highly-selective boron carrier that can deliver a high concentration of ^10^B into cancer cells, and concomitantly, a high tumor to normal tissue uptake ratio. In addition, cancer recurrence after BNCT treatment has significantly limited the clinical application of BNCT, and this could be attributed to the low boron content of small-molecule drugs, which are rapidly eliminated via blood circulation, and the lack of targeting ability of the boron drugs [14]. To overcome the disadvantages of small-molecule boron drugs, various approaches, such as the use of carborane and metallocarborane derivatives [15,16,17,18], macromolecules [19,20,21,22,23], dendrimers [24,25,26,27,28], liposomes [29,30,31,32,33,34,35,36], dextran conjugates [37,38,39], and polymer micelles [40,41] were employed to deliver a high loading of ^10^B to the tumor site with high specificity. These approaches utilize the multifunctionality of nanomedicine and the nanometer size of these nanostructures for accumulating in tumor site via the enhanced permeability and retention (EPR) effect. Among these third-generation boron drugs, boron-based nanostructures have emerged as a potential boron nanodrug for delivering a high loading of B-10 atom tumor site. The first demonstration of using boron nanoparticle instead of conventional soluble boron cluster as boron drug for BNCT was pioneered by Hokland in 2007. This early study showed that boron nanoparticle is non-toxic and can deliver a high loading of B-10 (~2000 ppm of boron) into the tumor cell [42]. This initial study showed an increase in survival rates and decreased in tumor growth after BNCT treatment. Hwang et al., later developed the most complex nanomedicine theranostic boron nanoparticle with silica shell, which enabled treatment of brain cancer via BNCT and image-guided delivery via optical and MRI [43]. However, further development of this [^10^B]-boron nanoparticles for BNCT is limited due to the low tumor to blood ratio. Various boron nitride nanomaterial has also been as boron drug delivery agent, due to the high loading of boron and simultaneously delivery anti-cancer drug selectively into the tumor cell [44,45]. However, surface functionalization is needed for the boron nitride nanomaterial due to the poor suspension in the biological solution [46,47,48]. Boron nitride nanotube (BNNT) functionalized with a biocompatible polymers showed three-folds greater in ^10^B accumulation compared to BSH in B16 melanoma cell [49,50]. Recent studies have also revealed that the cytotoxicity of boron-based nanomaterials increases with a decrease in the particle size, suggesting that the appropriate surface protection of boron-containing nanomaterial is essential for their development as potential nanotherapeutics [51]. Particularly, current approaches rely on the use of ^10^B-enriched boron precursor to ensure the sufficient loading of B-10 atoms into the tumor cells for an effective BNCT. However, the high time and cost consumption of ^10^B-enrichment process have limited further clinical application of BNCT. We hypothesized that the utilization of the natural abundances of ^10^B in boron-based nanomaterials and an appropriate surface functionalization is sufficient for achieving a potent anti-tumor effect via BNCT.

**Scheme 1:**
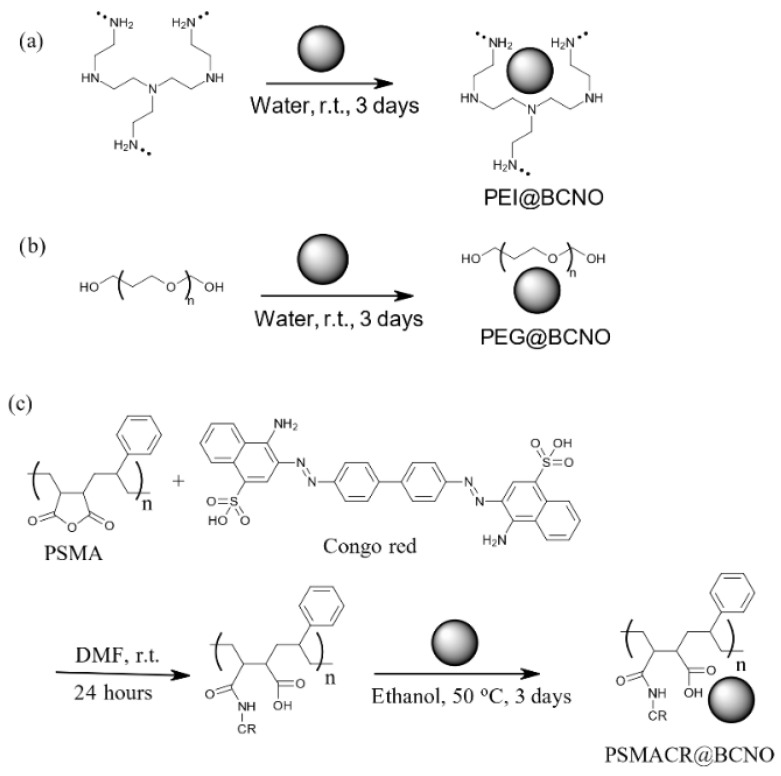
Schematic illustration of functionalization of BCNO nanoparticles with (**a**) PEI, (**b**) PEG, and (**c**) PSMACR ligands.

Herein, we demonstrate the synthesis and functionalization of non-^10^B enriched polymer-coated quasi-spherical boron carbon oxynitride (BCNO) nanoparticles as an effective boron drug for BNCT (Scheme 1). BCNO is a non-toxic, non-rare earth and earth-friendly phosphor with application in light emitting diodes (LEDs) [52,53,54,55,56], highly sensitive fingerprint readers [57,58], toxic metal sensors [57,59,60], and in high contrast cellular imaging [61,62]. In contrast to previously reported boron nanomaterials, BCNO possesses an inherent photoluminescence (PL) property without the need of an additional labelling chemistry for imaging purposes. For biomedical application, surface functionalization of nanomaterials is one of the utmost important criteria towards a successful treatment or imaging outcome. However, to date, only a few studies in functionalizing the surface of BCNO nanoparticles have been demonstrated [63,64]. Herein, we have successfully modulated the cytotoxicity, optical properties, cellular uptake and tumoricidal effect of the polymer-coated BCNO nanoparticles as a novel theranostic agent for BNCT. In contrast to previously reported boron nanomaterials, BCNO possesses an inherent photoluminescence (PL) property without the need of an additional labelling chemistry for imaging purposes. To achieve a successful BNCT, the deposition of a high concentration of ^10^B (20 µg/g of ^10^B per gram of tumor cells) within the tumor cell is required to induce a cytocidal effect in the tumor tissues. Conventionally, to ensure the sufficient loading of ^10^B within a tumor cell, ^10^B enriched precursor is utilized during the preparation of boron drug for BNCT. However, ^10^B enrichment process is a highly time-consuming and cost intensive separation process; thus, significantly limiting the development of boron drugs and the clinical application of BNCT. Recent work on polyboronic acid (PBA) nanoparticles prepared using non-enriched isotopes demonstrated promising results [65]. However, the PBA nanoparticles composed of a limited number of boron. As a result, the BNCT treatment efficacy using PBA was only comparable to ^10^B-BPA. In this study, we engineered non-^10^B enriched boron-based nanodrugs with higher tumoricidal effect than the state-of-the-art ^10^B-BPA treatment. Our results showed that through polymer functionalization, the cytotoxicity, cell uptake, tumoricidal effect and optical properties can be modulated to achieve a highly efficient and safe BNCT.

## 2. Materials and Methods

### 2.1. Chemicals and Instrument

Boric acid 99.99% (H_3_BO_3_) and hexamethylenetetramine ≥99% (C_6_H_12_N_4_) were purchased from Alfa Aesar (Lancashire, United Kingdom) as used without further purification. Guanidine hydrochloride 99.5% (CH_5_N_3_·HCl) was purchased from Arcos Organics. Polyethylene glycol (MW 20,000 g/mol) was purchased from Alfa Aesar without further purification. Polyethyleneimine (MW 1800 g/mol) was purchased from Alfa Aesar without further purification. Congo red was purchased from Alfa Aesar. Polystyrene maleic anhydride PSMA (MW 1900 g/mol) was purchased from Aldrich. Dimethylformamide (DMF) was purchased from Honeywell without further purification. The morphology of BCNO samples was analyzed via transmission electron microscope (JEOL, JEM-ARM200FTH, JEOL Ltd., Tokyo, Japan). Photoluminescence emission spectra were obtained on the photoluminescence spectrometer (PerkinElmer, LS55), and optical absorption spectra were determined by the UV-Vis spectrometer (HITACHI, U-3900). X-ray photoelectron spectroscopy was analyzed via high resolution X-ray photoelectron spectrometer (ULVAC-PHI, PHI Quantera II). Carbon correction was performed by using adventitious carbon at 284.6 eV. Infrared spectroscopy spectra were recorded by Fourier-transform infrared spectrometer (Bruker, Vertex 80v) using Attenuated Total Reflectance (ATR). XRD diffractions were obtained using the Bruker D2 spectrometer. Boron concentrations were measured via inductively coupled plasma optical emission spectrometer (ICP-OES) (Agilent 725). The ICP-OES boron content measurement of quasi-spherical BCNO nanoparticle was prepared by dissolving BCNO sample in 10 microliters of hydrofluoric acid (HF) and 1 mL of nitric acid (HNO_3_) and heated at 70 °C for 1 h. The 3-(4,5-dimethylthiazol-2-yl)-5-(3-carboxymethoxyphenyl)-2-(4-sulfophenyl) (MTS) reagent was purchased from Promega Cooperation and used as received.

### 2.2. Synthesis of BCNO Nanoparticle

BCNO nanoparticles were synthesized via a facile low-temperature sintering process. Boric acid (6 g, 0.1 mol), guanidine hydrochloride (3.09 g, 0.032 mol), and hexamethylenetetramine (0.46 g, 0.0032 mol) were dissolved in 100 mL of DI water and heated at 90 °C. Upon subsequent evaporation, a glutinous mixture was dried in an oven overnight to obtain a completely dried solid. The white solid was grounded into fine powder using a mortar and pestle and subjected to calcination in a tube furnace at 800 °C for 12 h under ambient atmosphere. After cooling to room temperature, a ‘yellowish’ solid was recovered (0.5 g). The as-prepared BCNO was purified by centrifugation with water and ethanol (the ratio of solvent and product was 10 mL:100 mg) at 6000 rpm for 10 min. After centrifugation, the product was redissolved in distilled water, diluted with ethanol (1:10 *v*/*v*), finally the BCNO solution was deposited onto carbon-coated copper grid for TEM analysis. For the XPS specimens, the purified BCNO sample was drop cast onto the silicon wafer.

### 2.3. Functionalization of the BCNO Nanoparticle with PEI and PEG Ligands

Functionalization of the BCNO nanoparticles was performed by dissolving the polymer ligands (PEG or PEI) into the BCNO nanoparticle solution in water with a mass ratio of 1:3. The mixture was then stirred at 25 °C for 3 days. The solution was then centrifuged at 6000 rpm for 10 min and repeated three times to obtain the isolated polymer-coated BCNO solid. Then the solid was re-dispersed in DI water at known concentration for further characterization.

### 2.4. Functionalization of the BCNO Nanoparticle with PSMA-CR

Functionalization of BCNO with PSMA-Congo red dye was performed in two steps. First, Congo red was substituted onto the anhydride moieties of PSMA via a base-catalyzed ring-opening substitution reaction [66]. The PSMA and Congo red dye with a mole ratio of 1:1 was dissolved in 20 mL of ethanol. Then, 5 mL of DMF were added into the solution as catalysts. The solution mixture was stirred for 24 h at room temperature. The mixture was concentrated by rotatory evaporator and precipitated in cold ether. Then the red color solid was dried in an oven at 80 °C for 12 h and stored at ambient conditions. The PSMA-CR was then functionalized onto the surface of BCNO nanoparticle via a ligand exchange reaction [67]. Briefly, the PSMA-CR (1 g) was first dissolved in ethanol (100 mL) and then added into a solution of 300 mg of BCNO nanoparticle in ethanol (100 mL) solution. The mixture was stirred at 50 °C for 3 days. The red solution was purified via centrifugation at 6000 rpm for 10 min and repeated three times to obtain the purified PSMA-CR coated BCNO nanoparticles as a red solid paste. The isolated solid (0.5 g) was re-dispersed in DI water at a known concentration for further characterization.

### 2.5. Synthesis of L-(4-^10^Borophenyl)alanine Fructose Complex (BPA-F)

BPA-F complex was prepared in a 1:1.1 weight percent ratio according to literature procedure [43]. In a typical experiment, 60 mg of ^10^BPA (98% ^10^B-enriched; Hammercap Medical AB, Stockholm) was dissolved in 10 mL of distilled water. To this turbid solution, 10 M NaOH solution was added dropwise to obtain a clear solution. Then, the addition of NaOH was continued until the pH reached ~10.5. After continuous stirring for 20 min, 66 mg of fructose was added and stirred for another 10 min. Then, the pH was adjusted to 8.0 with 12 M HCl, followed by the dropwise addition of 1 M HCl to obtain a solution with a pH of 7.4. Then, the BPA-F complex solution was filtered through a 1 µm and 0.45 µm filters and then stored at 4 °C overnight. After 24 h, the pH of the solution was re-measured at room temperature. Then, the solution was filtered again with 0.22 µm filter and finally stored at 2–8 °C.

### 2.6. Cell Viability Assay

The (3-(4,5-dimethylthiazol-2-yl)-2,5-diphenyltetrazolium bromide (MTT) assay was performed to evaluate the cytotoxic effect of BCNO and polymer-coated BCNO on ALTS1C1 cells. In a typical experiment, 1 × 10^7^ ALTS1C1 cells/mL were cultured and incubated for 24 h. After 24 h, the cells were removed from the culture plate by trypsinization. Then 5000 ALTS1C1 cells were added into a 96 well-plate along with 100 microliters of Dulbecco’s Modified Eagle Medium (DMEM). Then different concentrations of BCNO and polymer functionalized BCNO were added into each well and allowed to incubate with ALTS1C1 cells with different concentrations of BCNO solutions for 72 h. After 72 h of incubation, 10 microliters of MTT reagent (0.5 mg/mL) was added to each well and incubated for another 4 h at 37 °C. After 4 h of incubation with MTT reagent, the violet-colored cell pellet was dissolved in 1 mL of dimethyl sulfoxide (DMSO). The UV absorbances of the 96-well plate were recorded using an enzyme linked-immunosorbent assay (ELISA) plate reader at 570 nm. The absorbance values were then converted into cellular viabilities based on the calibration curve for different cell plating densities without any treatment of BCNO. Cell viability assay at 4 h was performed similarly by incubating the ALTS1C1 cells in 25 µg/mL concentration of bare BCNO, PEG@BCNO, PEI@BCNO and BPA-F complex for 4 h. The cell viability assays were performed similarly for the other boron drugs described in this study.

### 2.7. In Vitro Cell Uptake

In vitro cell uptake of BCNO and polymer-coated BCNO were performed by following a literature procedure [43]. Briefly, ALTS1C1 cells (2 × 10^4^ cells/mL) were cultured in a 6-well plate by placing a coverslip inside each well. After 24 h of incubation, the cells were attached to the surface of the plate. Then 0.05 mg/mL of BCNO and polymer-coated BCNO solution were added into each well and incubated for 4 h. The cells were then washed with PBS buffer followed by trypsinization and then resuspended in PBS and analyzed using flow cytometry under the FITC channel. The concentration of boron atom taken up by ALTS1C1 cells was determined via ICP-MS by the following procedure. Briefly, ALTS1C1 cells (2 × 10^4^ cells/mL) were cultured in a 6-well plate by placing a coverslip inside each well. After 24 h of incubation, the cells were attached to the surface of the plate. Then 0.05 mg/mL of BCNO and polymer-coated BCNO were added into each well and incubated for 4 h. The cells were then washed with PBS followed by trypsinization and then resuspended in PBS. Then the PBS suspension was centrifuged at 1500 rpm for 5 min, and the solid cell matrix was collected. The solid cell matrix was then dissolved in 10 microliters of hydrofluoric acid (HF) and 1 mL of nitric acid (HNO_3_) for ICP-MS boron measurement. Intracellular uptake of the bare BCNO and polymer-coated BCNO were imaged using confocal microscopy. First, the ALTS1C1 cells were cultured in a 6-well plate by placing a coverslip inside each well. After 24 h of incubation, the cells were attached to the surface of the plate inside each well. Then 0.05 mg/mL of BCNO containing solution were added to each well and incubated for 4 h at 37 °C. The cells were then washed with PBS three times, then the cells were stained with Fast dil and examined using confocal microscopy under the FITC channel to image the cellular uptake of the green fluorescence BCNO nanoparticles uptake into the ALTS1C1 cells. The in vitro intracellular boron uptake experiments were performed similarly using other boron drugs described in this study.

### 2.8. Thermal Neutron Irradiation Experiments

The thermal neutron irradiation experiments were performed by following a literature procedure [38]. Briefly 200 ul of DMEM medium solution containing 2 × 10^4^ of ALTS1C1 cells was loaded in the central 28-well of a 96-well plate. The plate was placed in an incubator at 37 °C to incubate for 24 h to allow the ALTS1C1 cells to stick on the bottom of the 96-well plate. After 24 h, the original DMEM medium solution were removed and 200 uL of DMEM medium solution containing 25 µg/mL of BCNO solution was added into each well. The plate was placed in the incubator for 4 h to allow for cellular uptake. After 4 h of incubation, the free BCNO nanoparticles in the solution were removed by washing the cells twice using PBS buffer solution. The plate was then irradiated by slow thermal neutron beam at the Tsing Hua Open Pool Reactor (THOR) for 15, and 30 min. After neutron irradiation, the plates were further incubated at 37 °C for 6 h, and then cell viability were determined and the cell viabilities were determined by MTS assay. MTS reagent (20 µL) of was added to each well and incubated for another 1.5 h at 37 °C. After 1.5 h of incubation with MTS reagent, the UV absorbances of the 96-well plate were recorded using an enzyme linked-immunosorbent assay (ELISA) plate reader at 490 nm. The cell viabilities of ALTS1C1 cells incubated with BCNO nanoparticles and the BPA-F complex after neutron irradiation were obtained by normalizing to the control group without BCNO nanoparticle and without neutron irradiation. All experiments were repeated five times and the average cell survival data are calculated. Thermal neutron irradiation experiments were performed similarly for the other boron drugs described in this study.

## 3. Results and Discussion

### 3.1. Synthesis and Characterization of Polymer-Coated BCNO

**Figure 1:**
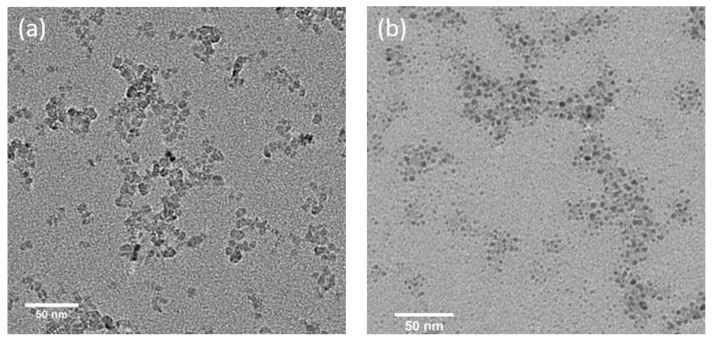
TEM images of (**a**) the as prepared BCNO nanoparticles and (**b**) PEG@BCNO after ligand exchange.

TEM images in Figure 1 revealed the as prepared BCNO nanoparticles is irregular in shapes with an average lateral diameter of 6.1 ± 0.73 nm) and possessed a turbostractic boron nitride diffraction patterns. (Figure 1a and Figure S1—XRD) The as prepared bare BCNO quasi-spherical nanoparticles and the PEG@BCNO nanoparticles remained dispersed in aqueous solution and randomly assembled onto the TEM Cu grid as shown in Figure 1. Upon ligand exchange, the size of the BCNO nanoparticles is slightly reduced to an average diameter of 5.1 ± 0.67 nm (Figure 1b) due to the effective surface passivation of the polymer ligands in stabilizing individual BCNO nanoparticles from coalescensing with neighboring BCNO as shown in Figure 1a. As a result, the aqueous dispersibility of the the polymer-coated BCNO nanoparticles is qualitatively more stable and the colloidal solution of the polymer-coated BCNO also remained stable for a prolonged period of time compared to the bare nanoparticles.

**Figure 2:**
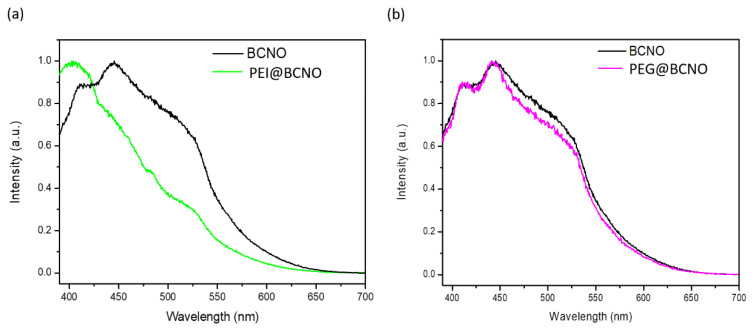
PL spectrum of the bare and functionalized BCNO upon photoexcitation at 365 nm of (**a**) stacked PL spectrum of quasi-spherical BCNO nanoparticles coated with PEI, bare BCNO (black trace), PEI@BCNO (green trace) (**b**) overlay PL spectrum of the bare BCNO (black trace) and PEG@BCNO (pink trace).

The photoluminescence (PL) spectra of the quasi-spherical BCNO nanoparticles with different polymer coating is presented in Figure 2. The PL spectra of the bare BCNO produced a broad emission with three bumps at 412 nm, 445 nm and 489 nm, which is consistent with literature reports [68,69]. Upon functionalization with PEG ligands, the photoluminescence spectra of PEG@BCNO remained the same compared to the PL spectra of bare BCNO nanoparticles. However, PEI@BCNO showed a dramatic 40 nm blueshift. Such as large PL spectra blue shift of PEI@BCNO can be explained by the high electronegativity of nitrogen acting as the electron donor ligand in inducing a higher energy state within the HOMO-LUMO energy gap of BCNO, as shown in the carbon-dot system [70,71]. As for PEG@BCNO, the oxygen atom on the polymer backbone served as both the electron donor and electron acceptor [71,72]. Such anomalies could explain for the lack of emission spectra shift of PEG@BCNO compared to the bare BCNO.

**Figure 3:**
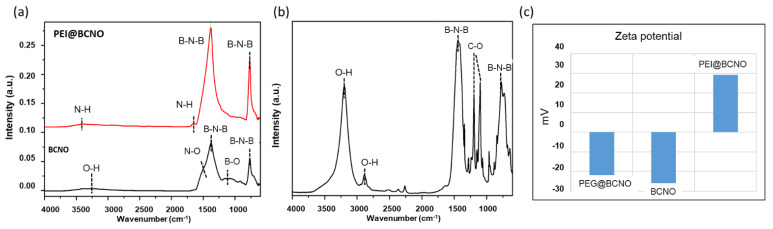
(**a**) FTIR spectrum of bare BCNO and PEI@BCNO (**b**) FTIR spectrum of PEG@BCNO (**c**) Zeta potential measurement of the surface charge of BCNO and polymer functionalized BCNO.

The FTIR spectra of bare BCNO nanoparticles and the polymer-coated BCNO is presented in Figure 3. The bare BCNO showed B-N in-plane stretching at 1400 cm^−1^, B-N out of plane stretching at around 900 cm^−1^, N–O stretching at around 1500 cm^−1^, a broad stretching of O–H group around 3200 cm^−1^, and a B–O stretching around 1200 cm^−1^. Such bonding characteristics confirmed the successful synthesis of BCNO [73,74]. Upon functionalization with PEI, there is a reduction in the B–O stretching, while the amine stretching intensity at around 1600 cm^−1^ and 3400 cm^−1^ increases. This result indicates that the B–O bonding was substituted with B-N bonding from the reaction between the amine group from PEI with the electrophilic boron on the surface of BCNO [75,76]. Upon functionalization with PEG, the C–O–C stretching at around 1200 cm^−1^ and C–O stretching at around 1100 cm^−1^ emerged, which corresponded to the ether functional group in PEG. The broad O–H stretching band at around 3400 cm^−1^ also significantly increases, which indicates that PEG was successfully functionalized onto the surface of the BCNO quasi-spherical nanoparticle [77]. Zeta potential measurements of the bare BCNO, PEG@BCNO, and PEI@BCNO is presented in Figure 3c. Both the bare BCNO and PEG@BCNO possessed negative surface charges of −29 mV and −27 mV, respectively. In contrast, PEI@BCNO possessed positive surface charge of 29 mV, due to the protonation of amine groups on the PEI (pKa = 8.2) at physiological pH. The change in the surface potential of PEI@BCNO implies the successful functionalization of PEI on BCNO.

**Figure 4:**
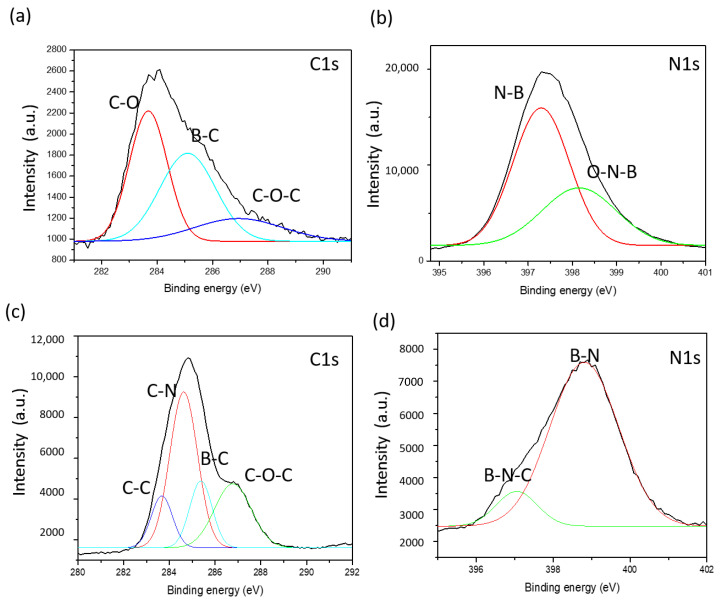
(**a**) XPS C1s spectrum of BCNO (**b**) XPS N1s spectrum of BCNO (**c**) XPS C1s spectrum of PEI@BCNO (**d**) XPS N1s spectrum of PEI@BCNO.

To further investigate the blue shift phenomena observed in PEI@BCNO, XPS analysis was conducted in order to elucidate the evolution of chemical bonding before and after functionalization with the nitrogen-containing ligand. XPS analysis of the bare quasi-spherical nanoparticle BCNO and PEI@BCNO are presented in Figure 4 and Table 1. The surface elemental composition of boron in PEI@BCNO was significantly reduced with concurrent increased in carbon content compared to the bare BCNO (Table 1). The high resolution C_1s_ spectrum of the PEI@BCNO is dominated by C–C and C–N bonding at 283.4 and 284.5 eV, respectively. (Figure 4a,b) Thirdly, the B-N-O peak in the N_1s_ spectrum of the bare BCNO was replaced by the B–N–C bonding at 396.8 eV upon functionalization with the PEI ligand (Figure 4b,d). Both the XPS and FTIR results further confirmed the successful functionalization of PEI polymer onto the surface of BCNO through a substitution reaction between the amino group of PEI and the B–O moieties on BCNO surface [76,78,79].

**Figure 5:**
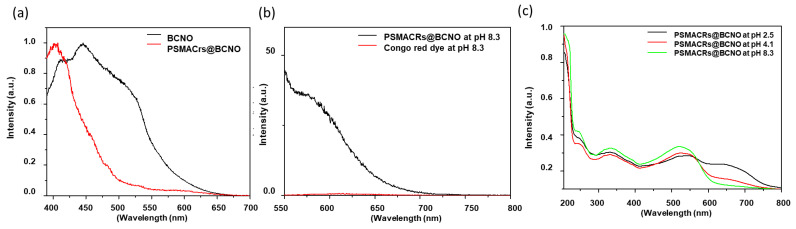
Photoluminescence spectrum of (**a**) BCNO (black trace) and PSMACRs@BCNO (red trace) upon excitation at 365 nm (**b**) Congo red dye only (red trance) and PSMACRs@BCNO (black trace) excited at 490 nm (**c**) UV-vis spectrum of the PSMACRs@BCNO at different pH values.

As a proof-of-concept, we demonstrated the functionalization of Congo red dye onto the surface of the quasi-spherical BCNO nanoparticles via a two-steps process. First, the amino group of the Congo red dye is substituted onto the electropositive anhydride moieties of the reactive PSMA copolymer. Subsequently, the Congo red substituted PSMA copolymer, abbreviated as PSMACR, is used to functionalize onto the surface of BCNO nanoparticles via a standard ligand exchange methodology [80]. In principle, this method can be extended to install other functionalities onto BCNO nanoparticles to impart desirable properties tailored for a specific application. Congo red is an organic red dye that is commonly used as a pH and chemical sensor [67,81,82], cell staining agent in biology, and as nano-theranostic agent [67,83,84,85,86]. It is also known that Congo red readily forms complex with amyloid fibril resulting in a phenomenon known as “aggregation-induced emission” (AIE). AIE refers to the enhancement in photoluminescence of Congo red upon self-aggregation, which has been utilized as biomarker for various amyloid-fibril related diseases, including Alzheimer’s disease [85,87]. The success of functionalization Congo red onto the surface of BCNO was further investigated using UV-vis and photoluminescence spectroscopy. Compared to the bare BCNO, the PSMACR@BCNO also showed a blue-shifted PL spectrum due to the electron donating effect of the amine group of CR.(Figure 5a) Furthermore, the emission band at 600 nm of PSMACR@BCNO was significantly enhanced compared to free Congo-red dye due to the AIE phenomenon. (Figure 5b red trace) The AIE behavior observed in PSMACR@BCNO could be explained by two folds. First, the confinement of PSMACR polymer ligand onto the surface of BCNO increases the local concentration of the CR dye. The increased in local centration of CR dye on the surface of the BCNO nanoparticles leads to the self-aggregation of CR red through their hydrophobic interactions, giving rise to the AIE phenomena. Furthermore, UV-Vis spectrum of the purified PSMACR@BCNO showed signature absorbance bands from both BCNO and Congo red in water with maxima at 240, 335, and 493 nm.(Figure 5c) The 240 nm absorbance could be assigned to the electronic transition from the valence band to the nitrogen vacancy level in BCNO and from the π–π* transition of the aromatic group in CR. The 335 nm and 493 nm absorbance bands could be attributed to the π–π* transition of the –NH and the π–π* transition of the azo group in CR, respectively [88,89]. In addition to the AIE behavior of CR, CR also exhibits pH dependence optical behavior. When the pH of the solution is above the pKa of the Congo-red dye (pKa ~ 5.5), the disulfonate dianion of Congo red gives rise to the absorbance band at 335 and 493 nm. At a lower pH value, the nitrogen and azo bond are protonated resulting in the emergence of a new absorbance band at 653 nm. Both the UV-vis absorbance and photoluminescence spectra confirmed that PSMACR@BCNO retained their photophysical properties of Congo red upon functionalization onto BCNO nanoparticles. This proof-of-concept demonstration enables BCNO to be functionalized with various chemical moieties for a wide range of applications, including as biomarker, nanotheranostic agent, hybrid organic-semiconductor devices, and pH chemical sensors [67,82,86,90].

### 3.2. Cytotoxicity Study of the BCNO, PEI@BCNO, and PEG@BCNO

**Figure 6:**
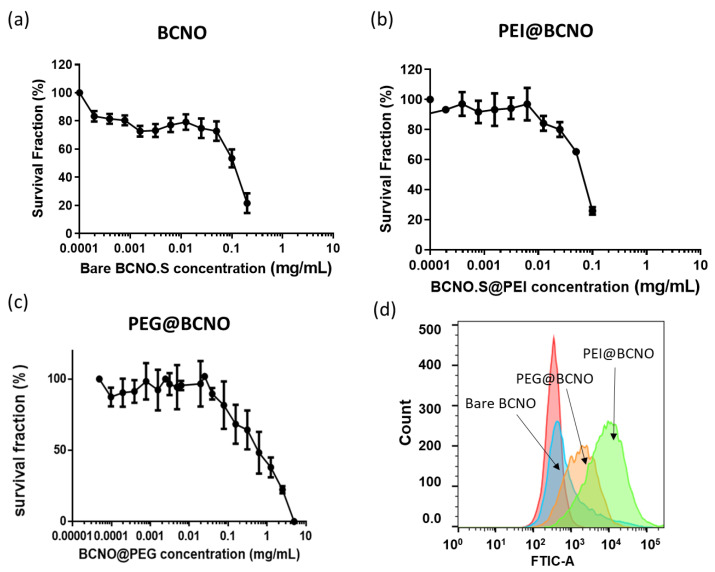
(**a**) MTT assay of BCNO on ALTS1C1 (**b**) MTT assay of PEI@BCNO on ALTS1C1 (**c**) MTT assay of PEG@BCNO on ALTS1C1 (**d**) flow cytometry of bare BCNO, PEI@BCNO and PEG@BCNO.

The boron drug must have low cytotoxicity and high biocompatibility (i.e., dispersion in aqueous media) to be developed as a safe and effective BNCT drug. The as-prepared BCNO was readily dissolved in water, and it exhibited a concentration-dependent cytotoxicity behavior in the astrocytoma ALTS1C1 cells [43,91]. Based on previous work on the preparation of ^10^B-enriched boron nanoparticles for treating murine brain tumors via BNCT, we used the ALTS1C1 cells as a model study for a proof-of-concept demonstration [43]. Although it is well known that nanoparticle drugs cannot infiltrate the blood–brain barrier (BBB) to treat brain-related diseases, researchers have used various strategies to temporarily disrupt the BBB to allow nanotherapeutics to be delivered to the brain [92,93]. Moreover, research has demonstrated that the ALTSIC1 murine brain tumor model is highly infiltrative, making it a suitable candidate for future in vivo studies [91]. After 72 h of incubation, the half-maximal inhibitor concentrations (IC50) of the bare BCNO, PEG@BCNO, and PEI@BNCO were determined to be 0.1 mg/mL, 0.3 mg/mL, and 0.05 mg/mL, respectively.(Figure 6a–c) As anticipated, the bare BCNO nanoparticle exhibited higher cytotoxicity than PEG@BCNO because of the production of reactive oxygen species (ROS) on the surface of the BCNO nanoparticles, which resulted in oxidative stress of the cells [51,94,95]. Conversely, the positively charged PEI@BCNO exhibited the highest cytotoxicity among the three BCNO samples investigated in this study, because of the cationic PEI ligand’s ability to induce apoptosis and cell necrosis in the targeted ALTS1C1 cell [96,97]. One of the greatest limitations of small molecule drugs, such as BPA, is the requirement for continuous BPA infusion in the blood to maintain BPA accumulation in tumor cells. Therefore, the concentration of ^10^B in the blood is also high, posing significant health risks to the patient during BNCT. According to the literature, nanodrugs are actively internalized through the formation of multiple interactions with the cell membrane, resulting in nanoparticle endocytosis and remaining trapped within the cell [65,98]. More in-depth in vivo biodistribution studies will be conducted to confirm the advantages of boron-based nanodrugs for BNCT over small molecule BPA. Cell internalization can be enhanced through appropriate surface functionalization with specific ligands that target the overexpressed receptors on cell membranes or with positively charged polymer ligands that can interact favorably with negatively charged glycocalyx. Based on these findings, the positively charged PEI@BCNO did have the highest cell internalization, followed by PEG@BCNO, while the bare BCNO had the lowest cell internalization, as confirmed via flow cytometry analysis (Figure 6d). Effective cell internalization and accumulation are essential for achieving effective BNCT using non-^10^B-enriched precursors.

**Figure 7:**
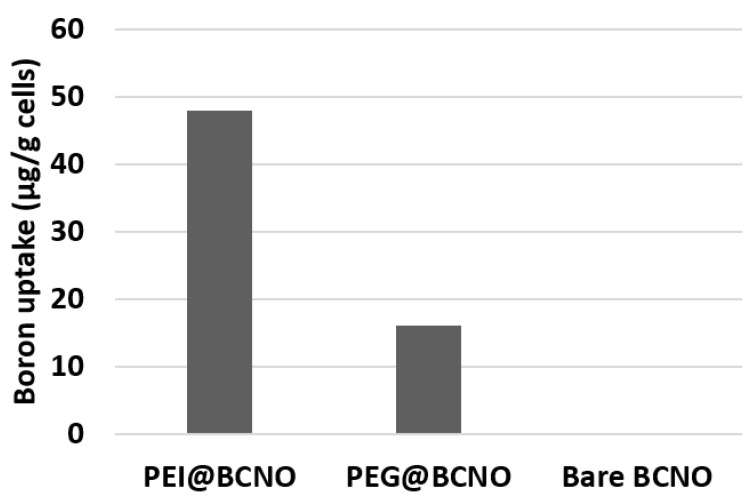
ICP-MS cell uptake of PEI@BCNO, PEG@BCNO and bare BCNO with ALTS1C1 cells.

Compared to small molecule boron drug, which contained one boron atom per molecule, the as prepared BCNO quasi-spherical nanoparticles contained about 30% of boron atom per nanoparticles based on ICP-OES analysis. We hypothesized that a rationally engineered BCNO nanoparticles could deliver sufficient ^10^B for effective BNCT treatment by taking advantage of the natural abundances of ^10^B isotopes (~80% and ~20% of ^11^B and ^10^B) and the improved cell internalization characteristic. To test this question, the intracellular uptake of the bare BCNO, PEG@BCNO and PEI@BCNO into ALTS1C1 cells was measured via ICP-MS to be negligible, ~16 µg and 48 µg of boron/g of cell, respectively (Figure 7). This proof-of-concept demonstration showed that the cytotoxicity and cell internalization capability of BCNO can be simply modulated by functionalizing their surfaces with appropriate polymers, opening up opportunities of advancing BNCT research in using non-^10^B enriched precursor.

**Figure 8:**
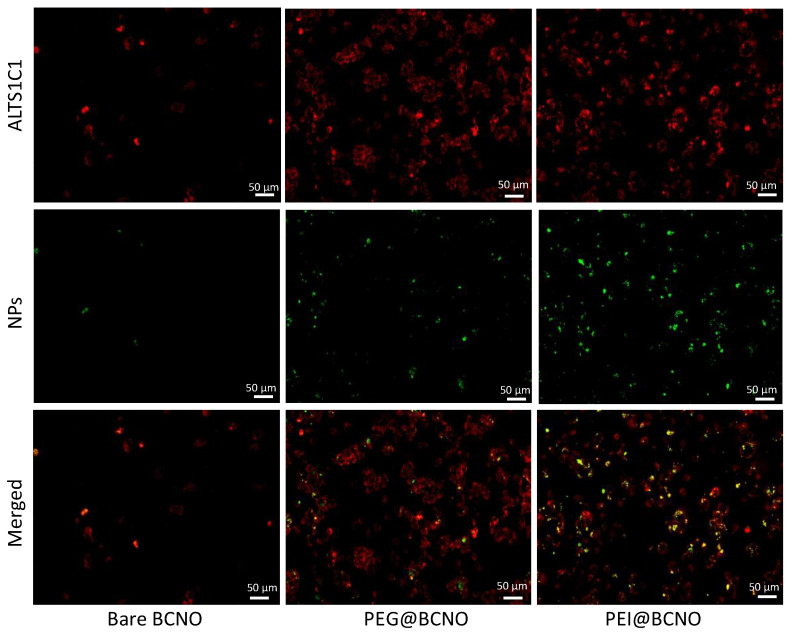
Fluorescence microscopy images of bare BCNO, PEG@BCNO and PEI@BCNO incubated with ALTS1C1 cells for 4 h. The red fluorescence corresponds to the ALTS1C1 cells stained with Fast-dil, while the green fluorescent correspond to the BCNO nanoparticles. Scale bar: 50 µm.

Taking advantage of their inherent photoluminescence properties of BCNO nanoparticles, along with their high boron content, the distribution of polymer-coated quasi-spherical BCNO nanoparticle could be detected using a fluorescence microscope [61,62]. The ALTS1C1 cells were stained with Fast-dil dye, which appeared red, while BCNO nanoparticles showed green fluorescence upon photoexcitation under the FITC channel. Intracellular colocalization of BCNO nanoparticles and the ALTS1C1 cells appeared as yellow fluorescence in the merged image, as shown in Figure 8. Qualitatively, the cell uptake of the PEI@BCNO is much higher compared to the PEG@BCNO and the bare BCNO nanoparticles. The fluorescence microscopy results support the flow cytometry experiments, which concluded that cell uptake efficiency increases from the bare BCNO, PEG@BCNO and PEI@BCNO, respectively.

### 3.3. In-Vitro Boron Neutron Capture Therapy Using Polymer-Coated BCNO Nanoparticles

**Figure 9:**
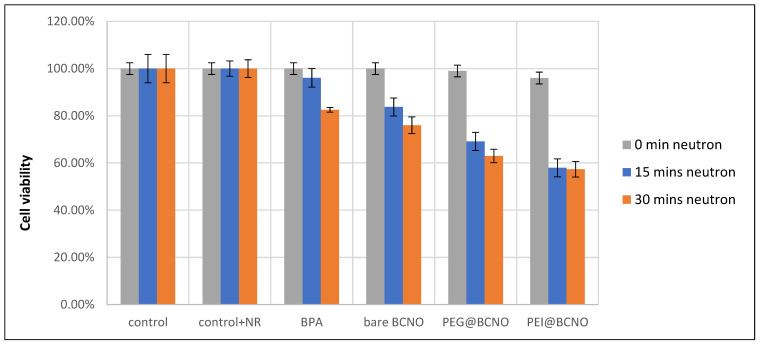
Normalized cell viabilities of ALTS1C1 cells as a function of neutron irradiation dose and BCNO nanoparticles. The ALTS1C1 cells were internalized with bare BCNO, PEG@BCNO and PEI@BCNO nanoparticles. The cell viability was measured at 6 h after neutron irradiation. The error bar represents the standard deviation of five independent measurements.

To test our hypothesis that non-^10^B-enriched polymer-coated BCNO nanoparticles can exhibit a tumoricidal effect via BNCT, in vitro cellular neutron flux experiments were conducted using ALTS1C1 cells. A fixed concentration of 25 µg/mL dosage of the BCNO nanoparticles and BPA-fructose complex was used for the thermal neutron irradiation experiment, and the cellular cytotoxicity was low after 4 h of incubation. Before neutron irradiation, the ALTS1C1 cells incubated with BPA, bare BCNO, and PEG@BCNO showed negligible cell death, whereas PEI@BCNO showed ~3% of cell death, according to the cell viability analysis (Figure 9). The neutron flux experiments were conducted using the Tsing Hua Open-Pool Reactor (THOR) at Hsinchu, Taiwan. In our study, we chose a thermal neutron beam intensity of 1 × 10^9^ n/cm^2^ s based on the literature [43]. ALTS1C1 cells incubated with BCNO nanoparticles were exposed to thermal neutron irradiation for 15 and 30 min, with a neutron beam intensity of 1 × 10^9^ n/cm^2^ s. The optical densities were normalized to the control set of experiments without nanoparticle incubation and thermal neutron irradiation, and the cell viabilities were obtained using the MTS assay. After thermal neutron irradiation, the ALTS1C1 cells that were internalized with the bare BCNO, PEG@BCNO, and PEI@BCNO had acute cell death compared to the controlled group (Figure 9). More cell deaths were observed when the neutron irradiation time was increased from 15 to 30 min for all the ALTS1C1 cells internalized with BCNO nanoparticles. Remarkably, our study showed that the non-^10^B-enriched bare BCNO, PEG@BCNO, and PEI@BCNO caused acute cell deaths of 24%, 37%, and 43%, respectively, after 30 min of neutron irradiation (27 × 10^11^ n/cm^2^ neutron dose). Compared to the state-of-the-art ^10^B-enriched BPA-F complex treatment, only 17% of cell death was measured. Although the ALTS1C1 cells showed negligible intracellular boron uptake (Figure 7), the tumoricidal effect of the bare BCNO nanoparticles on the ALTS1C1 cells can be explained by the slight differences in sample preparation for the intracellular boron uptake and neutron flux experiments. According to the neutron flux experiment protocols, residual-free BCNO nanoparticles could remained on the well plate, which was subsequently irradiated with a slow thermal neutron beam. Thus, the residual BCNO nanoparticles that remained on the plate produced LET particles during the thermal irradiation, inducing a moderate cell-killing effect. Since LET particles have a path length of 5–9 µm, cells within the ionizing radiation path will be affected. Furthermore, the slight differences between the tumoricidal effects of PEI@BCNO and PEG@BCNO despite the large differences in the intracellular boron uptake (Figure 7) can be explained by the exocytosis mechanism of nanoparticles. Since the cell viability analysis was conducted 6 h after neutron irradiation, there is a high probability that the PEI@BCNO nanoparticles were pumped out from the ALTS1C1 cells via the exocytosis mechanism during the 6 h incubation period. This could also explain the small differences in the cell viability of the ALTS1C1 cells upon being treated with PEI@BCNO and PEG@BCNO. In future studies, more investigations will be conducted to clarify this hypothesis. Nevertheless, we showed that the non-^10^B-enriched PEG@BCNO and PEI@BCNO exhibited effective tumoricidal effects because of the enhanced intercellular localization achieved through polymer functionalization and the high boron content in the BCNO nanoparticles. This proof-of-concept demonstration created an opportunity for a new economical approach toward designing a safer and more potent boron nanodrug for BNCT application.

## 4. Conclusions

In conclusion, we have successfully demonstrated an innovative approach in developing a non-^10^B enriched boron nanodrug for BNCT with potent tumoricidal effect. Through polymer functionalization of the quasi-spherical BCNO nanoparticle with PEI and PEG polymer ligands, their tumor killing effect, cytotoxicity, cell uptake, and optical properties can be modulated. The PEI@BCNO was engineered to possess a positive charge surface potential on the BCNO nanoparticle and thus demonstrated a higher in vitro cell penetration (~48 µg B/g of cell), while the PEG@BCNO nanoparticles exhibited significantly reduction in cell cytotoxicity and with moderate intercellular cell uptake (~16 µg B/g of cell). An in vitro neutron flux experiment confirmed that the non-^10^B enriched polymer-coated BCNO nanoparticles resulted in 24%, 37% and 43% of acute cell death for ALTS1C1 incubated with bare BCNO, PEG@BCNO and PEI@BCNO, respectively. Under similar neutron dose irradiation and nanoparticles dosing, this study demonstrated higher tumor killing effect compared to the state-of-the-art boron nanodrug and the BPA-F molecular drugs (13% cell death). In vivo BNCT studies will be performed to evaluate the tumoricidal effect of polymer-coated BCNO nanoparticles and their biodistribution in mice. This research could potentially pave a new and economical approach towards designing a safer and more potent boron nanodrug for BNCT application.

## Figures and Tables

**Table 1:** XPS surface elemental composition of BCNO and PEI@BCNO.

Sample	B	C	N	O
BCNO	40.6%	7.6%	36.3%	15.5%
PEI@BCNO	3.4%	54.9%	25.8%	16.8%

## Data Availability

The data is available on reasonable request from the corresponding author.

## Supplementary Materials

The following are available online at https://www.mdpi.com/article/10.3390/nano11112936/s1, Figure S1: XRD pattern of the as prepared BCNO displaying a broad peak ca. 26.6° and another broad peak at 43.1°, which represents the (002) plane reflection of hexagonal boron nitride(h-BN) and the unresolved reflection planes of h-BN, respectively.
