# Supplementary material for: Polymer-Coated Nanoparticles for Therapeutic and Diagnostic Non-10B Enriched Polymer-Coated Boron Carbon Oxynitride (BCNO) Nanoparticles as Potent BNCT Drug
Source: Nanomaterials (Basel). 2021 Nov 2;11(11):2936. doi: 10.3390/nano11112936 (PMC8618246; doi:10.3390/nano11112936)

Polymer-Coated Nanoparticles for Therapeutic and Diagnostic Non-^{10}B Enriched Polymer-Coated Boron Carbon Oxynitride (BCNO) Nanoparticles as Potent BNCT Drug

Chen-Wei Chiang [1], Yun-Chen Chien [1], Wen-Jui Yu [2], Chia-Yu Ho [1], Chih-Yi Wang [1], Tzu-Wei Wang [1], Chi-Shiun Chiang [2] and Pei-Yuin Keng [1,*]

Supporting Information

Figure S1: XRD pattern of the as prepared BCNO displaying a broad peak ca. 26.6° and another broad peak at 43.1°, which represents the (002) plane reflection of hexagonal boron nitride(h-BN) and the unresolved reflection planes of h-BN, respectively.

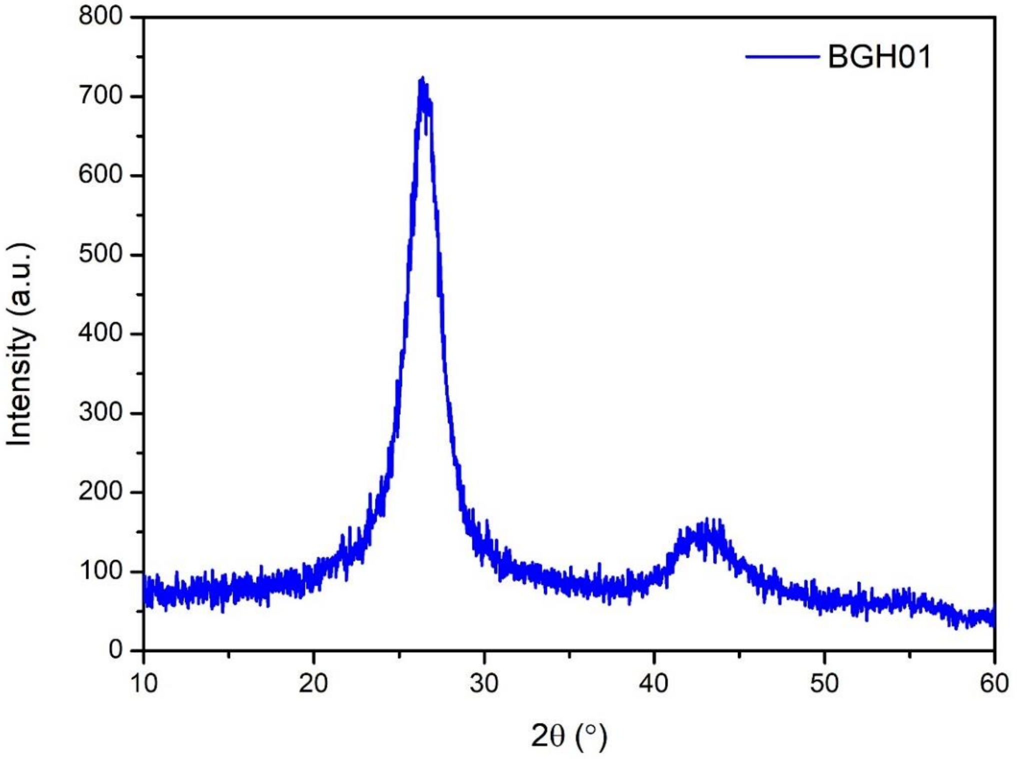

Supplement: Supplementary file 1 [file nanomaterials-11-02936-s001.zip › nanomaterials-1407018-supplementary.pdf]
